# What Are You or Who Are You? The Emergence of Social Interaction between Dog and an Unidentified Moving Object (UMO)

**DOI:** 10.1371/journal.pone.0072727

**Published:** 2013-08-28

**Authors:** Anna Gergely, Eszter Petró, József Topál, Ádám Miklósi

**Affiliations:** 1 Department of Ethology, Eötvös Loránd University, Budapest, Hungary; 2 Institute of Cognitive Neuroscience and Psychology, Hungarian Academy of Sciences, Budapest, Hungary; 3 MTA-ELTE Comparative Ethology Research Group, Budapest, Hungary; Institut Pluridisciplinaire Hubert Curien, France

## Abstract

Robots offer new possibilities for investigating animal social behaviour. This method enhances controllability and reproducibility of experimental techniques, and it allows also the experimental separation of the effects of bodily appearance (embodiment) and behaviour. In the present study we examined dogs’ interactive behaviour in a problem solving task (in which the dog has no access to the food) with three different social partners, two of which were robots and the third a human behaving in a robot-like manner. The *Mechanical UMO* (Unidentified Moving Object) and the *Mechanical Human* differed only in their embodiment, but showed similar behaviour toward the dog. In contrast, the *Social UMO* was interactive, showed contingent responsiveness and goal-directed behaviour and moved along varied routes. The dogs showed shorter looking and touching duration, but increased gaze alternation toward the *Mechanical Human* than to the *Mechanical UMO*. This suggests that dogs’ interactive behaviour may have been affected by previous experience with typical humans. We found that dogs also looked longer and showed more gaze alternations between the food and the *Social UMO* compared to the *Mechanical UMO*. These results suggest that dogs form expectations about an unfamiliar moving object within a short period of time and they recognise some social aspects of UMOs’ behaviour. This is the first evidence that interactive behaviour of a robot is important for evoking dogs’ social responsiveness.

## Introduction

The behaviour ecological approach defines social behaviour as interactions between individuals of the same species that has fitness consequences [1], and which, at the functional level, is organised for achieving different goals such as finding a suitable mate, evading predators, cooperating in the acquisition of food etc. Social behaviour has evolved specifically to contribute to the survival of the individual if group living provides some selective advantage. Because of the functional similarities in the life of different species one may expect that a range of social behaviours reflect some commonalities (matching competencies) based on ancient homologies or convergent evolutionary processes. Given that group living or limited co-existence may also confer some advantages in the case of different species social behaviour could also emerge in heterospecific context, both developmentally and on an evolutionary time scale (e.g. interspecific communication, see also [2], [3]). One well known example for this is the collaboration between honeyguide birds *(Indicator indicator*) and African tribal people in order to find honey by locating beehives in the forest [4]. In another case Bshary et al show that the grouper (*Plectropomus pessuliferus*) and the giant moray eel (*Gymnothorax javanicus*) hunt cooperatively, probably, because they have complementary behavioural skills, and the two partners, belonging to different species, are able to coordinate their actions at the behavioural level, that is, the grouper uses a specific visual signal to lure the moray eel on a hunting trip [5].

Investigating social behaviour of animals living in groups by the means of controlled experiments is essential in the study of animal behaviour. However, the nature of social interactions makes experimental investigations very difficult due to many different reasons. First, the behaviour of the individuals is dependent on their interaction partners. Second, it is nearly impossible to manipulate and control behaviour of a living individual for longer duration, and third the interaction is always influenced by prior experiences related to participating individuals (see also [6]).

One solution to these problems has been to use artificial stimuli or stimulus objects that resembled to different degree conspecific companions. For example, in the early years of ethology Tinbergen [7] used this method to evoke social behaviour (e.g. courtship or territorial behaviour) in different animal species (e.g. sticklebacks – *Gasterosteus aculeatus*). The use of more or less schematic models in a systematic way allowed researchers to determine which properties of the stimulus act as behavioural releasers (cf. sign stimulus) and have the potential to evoke particular behaviour (cf. modular action patterns) which are comparable to that observed under natural conditions (e.g. [8], [9]). Nowadays behaviour biologists and engineers are developing more complex models, autonomous or remote controlled devices, which are able to stimulate subject animals.

This trend has become even more popular with the possibility to construct more sophisticated stimuli, “robots” [10]. Krause et al [6] argued that using such artificial agents (robots) as social partners could enhance controllability and reproducibility in the experimental techniques. Thus many researchers use now robots for studying “intra-specific” social interactions (e.g. [11]–[14]).

However, the utilisation of robots confers also further advantages, that is, it is possible to separate experimentally the effects of bodily appearance (embodiment, cf. [6], [15]) and behaviour [6]. This approach has been utilised particularly in cognitive and developmental psychology, in order to find out whether, from the infant’s perspective, humans have any advantage over non-human artifacts (machines) if they act in the similar way. In his classic study Meltzoff [16] reported that 18-month-old infants imitated the movements of a human hand but failed to replicate the same movement when it was executed by a robotic “hand”. He argued that the infants at this age are attributing intentions to humans but not to non-human agents. In a later study Meltzoff and co-workers [17] demonstrated that 18-month-old infants follow more likely human-like robot’s gaze if they saw it act in social-communicative fashion, thus the emergence of social interaction depends also on experience.

In the past 10 years many robots have been used to investigate social behaviour in animals. The common feature of these approaches was that the investigators wanted to make the robot as similar as possible to the species studied [6]. For example, Kubinyi and her colleagues [18] investigated dogs’ social behaviour toward a dog-like robot (AIBO) and showed that the dogs’ age, the experimental context and external features of the AIBO had an effect on dogs’ behaviour. In another study dogs encountered a life sized dog model which had either a short or a long, wagging or not wagging tail. Dogs approached more likely the long-tailed model if it was wagging the tail [19].

The conceptual separation of behaviour and cognition (mind) from the body has a long history in the cognitive sciences (e.g. [15]), with the general insight that cognition is not possible without a body [20]. This theoretical issue could be put to test in several forms, given the advance in technology. One important question could be whether animals (or humans) are able to recognise and react to behaviour patterns independently from the embodiment. This approach opens ways for experimenting in which researchers look at the extent and limitation (both on the part of the observer and the agent) to engage in social interaction. Such data would be important to reveal the flexibilities of animal and human mind, including evolutionary and developmental factors.

Using artificial agents in a social context may reveal the animals’ ability to recognise some aspects of the other’s behaviour and the quality and quantity of experience needed for such recognition to emerge and/or to get improved. As far as we know, however, such approach, in which the embodiment and the behaviour of the agent are varied in a systematic way, has not yet been utilised in animals. Importantly, in this case the embodiment should be as distinct as possible from the range of objects with which the subject interacts in a social way under habitual (natural) conditions. Such investigations can have specific significance when one wants to understand the mental aspects of some complex social behaviour such as social learning or intentional communication. The critical feature of this approach is the utilisation of an unfamiliar object that is able to execute actions in different manners. In principle this agent can take any form and shape, so we would introduce the general term of an unidentified moving object (UMO) which emphasises that at the time of the first encounter the animal subject has no previous experience with that particular artificial agent. The overall goal of such experiments is to find out under which conditions is the subject able to interact with the UMO given the possibility that both the embodiment and the behaviour can be modified, and interactions can be repeated both in space and time.

This study has been designed to provide a proof of this concept. We decided to use dogs as subjects, especially because they are becoming very popular in studying complex social behaviours. Dogs may also be favourable subjects for these studies because they have shared a common environment with humans (a heterospecific agent) for a long time, and they live also in human families at present. Thus dogs may be especially skilful at interacting with non-dog-type agents (UMOs) if they can recognise some aspects of the behaviour of those agents.

The method of the present study originates from the well-documented observations on communicative interactions between dogs and humans in problem solving situations (for details see [21]–[23]). In these scenarios a human hides a piece of food in the presence of a dog at an inaccessible location. After the departure of the hider the dog has the opportunity to interact with a naive human (owner) entering the room for a short time. The original experiment [21] involved also two control conditions in which dogs were left alone after the hiding or no food was hidden. Dogs seemed to utilize both gazing and gaze alternations between the place of food and the owner during the interaction and these behaviours were more frequent in the presence of the owner and hidden food than in the absence of a human or when no food had been hidden. In most cases dogs were also successful to direct the naive human to the place of the hidden food (see also [24]).

Based on these findings, we aimed to compare how adult pet dogs perform in an analogous problem solving task with different partners. There are three different partners: ‘mechanical’ or ‘social’ UMOs and a ‘mechanical’ human (see below). Using a between-subject design we compare the emergence of dogs’ social and communicative behaviours toward the different partners. We endowed the social UMO with different external (eye spots) and internal (goal directedness, interactive responsiveness, varied movements) properties that are general characteristics of entities with minds (people or animals) to which infants may be sensitive (for a review see [25]). We have hypothesised that dogs would display similar behaviour toward the mechanical partners (UMO and human). At the same time they are expected to increase their social behaviours toward the social UMO after repeated encounters, which would indicate that they are able to recognise some aspects of UMOs’ social behaviour.

## Materials and Methods

### Ethic Statement

Our experiment is based on non-invasive procedures for assessing dogs’ behaviour. Non-invasive studies on dogs are currently allowed to be done without any special permission in Hungary by the University Institutional Animal Care and Use Committee (UIACUC, Eötvös Loránd University, Hungary). The currently operating Hungarian law “*1998. évi XXVIII. Törvény” -* the Animal Protection Act – defines experiments on animals in the 9^th^ point of its 3^rd^ paragraph (3. §/9.). According to the corresponding definition by law, our non-invasive observational study is not considered as an animal experiment.

**Figure 1:**
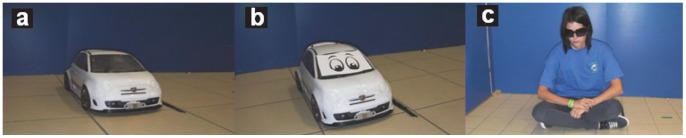
The three test partners: a; *Mechanical UMO* b; *Social UMO* c; *Mechanical Human* (for more details see text).

The owners responding to our advertisement at the department’s homepage (http://kutyaetologia.elte.hu) volunteered to participate. The woman pictured in Figure 1 and subjects in Video S1 have given written informed consent, as outlined in the PLOS consent form, to publication of their photograph or video.

### Subjects

50 adult pet dogs were recruited from the Family Dog database of the Department of Ethology, Eötvös Loránd University. We excluded 3 dogs because they displayed high level of anxiety-related behaviours in the experimental room (N = 2) or upon encountering the UMO (N = 1). The remaining 47 dogs were assigned to one of three experimental conditions: *Mechanical UMO* (N = 15, 5 males, 10 females, mean age±SD: 3.6±2.3 years), *Social UMO* (N = 17, 9 males, 8 females, mean age±SD: 4.6±3.2 years) and *Mechanical Human* (N = 15, 7 males, 8 females, mean age±SD: 3.7±3.2 years). Only dogs older than 1 year were recruited, and there was no upper age limit to participate. Therefore some old dogs (older than 10) were also included and this increased the age range. Importantly, however, our analysis of the dogs’ mean age did not show significant differences between the 3 groups (One-way ANOVA p = 0,607, F_2,44_ = 0.504). Subjects were allowed to participate only if they could be motivated with food. Each subject participated only in one condition.

### Apparatus

**Figure 2:**
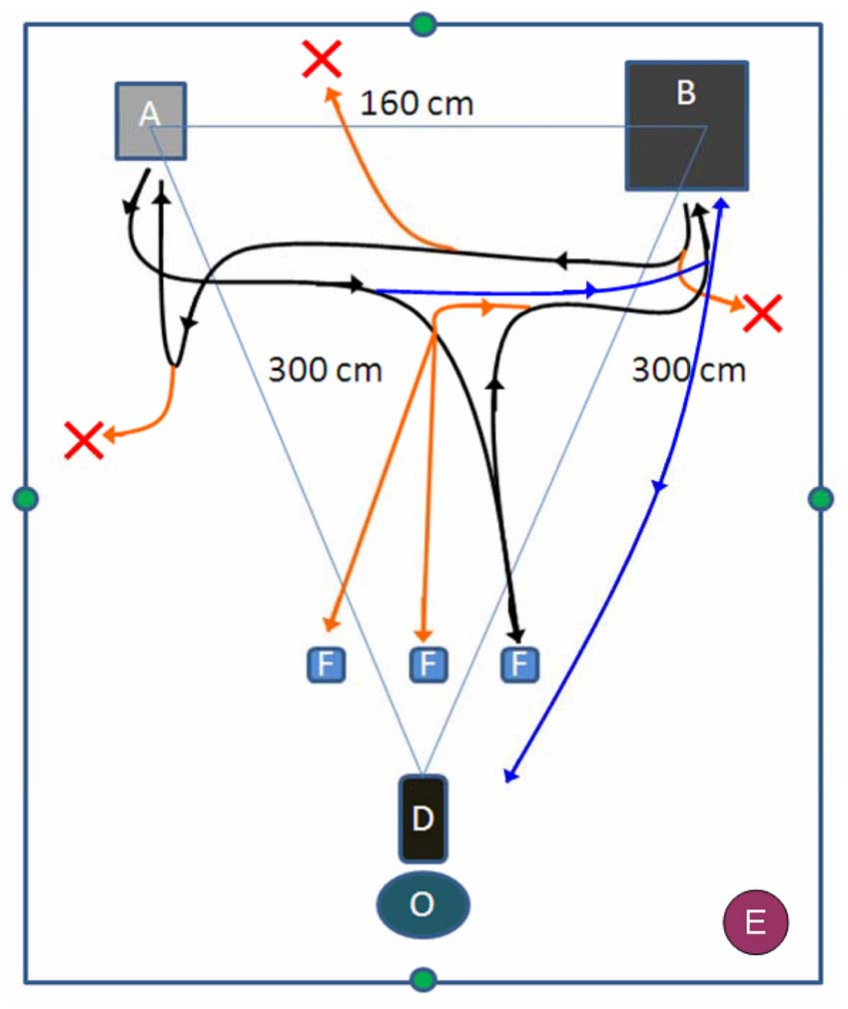
Experimental room and paths of partners’ move. O = place of the owner, D = place of the dog, E = position of Experimenter 2, F = three plates as potential food sources, A = start point of the partner, B = place of the box. Green circles indicate the location of the cameras. The triangle presents distance between the dog the partner and the place of the inaccessible food (box). Black lines show the paths of the partner to the plate (location of the food), to the box and back to the start point. Orange lines show the different path of the *Social UMO* compared to the Mechanical partners (UMO or Human) to each plates, box and different start points during the 2^nd^ to 6^th^ trials (red X). Blue lines show the path which in the partner goes back to the box from the start point and bring the food to the dog.

Dogs were tested at the Department of Ethology, Eötvös Loránd University in a 4.5 m×3.5 m test room. In this experiment we used a remote-controlled (RC) car (#32710 RTR SWITCH, 28 cm x 16 cm x 13 cm) as UMO which was supplemented with two magnets on its back and front. The car was controlled by Experimenter 2 (E2), who was standing in the corner of the lab (see Figure 2). Throughout the experiment she avoided carefully getting engaged with the dog.

A metal wire mesh box (61 cm x 46 cm x 54 cm) was used as a hiding location, with a fixed magnet inside, and three transparent plastic bowls (10 cm×10 cm) were used as potential food sources, one was equipped with two metal sheets. We recorded each trial with four cameras in the test room (see Figure 2).

Three magnets with different strength were used in the experiment. The weakest magnet was placed on the front of the car (UMO) which was supposed to connect to one of the metal sheets on the bowl with the food. Hence the UMO carried the food into the box that was now inaccessible for the dog. The moderately strong magnet was placed inside the box. It was supposed to attach to the other metal sheet on the bowl when the UMO transported the bowl into the box. Thus the UMO was “able to” leave the food inside the box. The most powerful magnet was placed on the back of the UMO. This was used when the UMO reversed into the box in order to carry the food to the dog.

### Test-partners

In the *Mechanical UMO* and *Social UMO* conditions we used the same RC car as a partner. However, the *Mechanical UMO*, moved always along the same path during the experiment, and approached the plastic bowl always from the same location.

In contrast, the *Social UMO* had two eye spots (2 cm in diameter, placed on the engine hood) (see Figure 1), and it moved along varied paths in the room during the experiments, it went to different start points in the lab, approached both empty and baited bowls (“made a choice” see below), and started to move when the dog looked at it in particular situations (responded to dog’s behaviour) (for details see Procedure). In order to control for the embodiment we included a *Mechanical Human* condition in which a female human was the partner. We wanted to make her behaviour highly similar to that displayed by the *Mechanical UMO.* She was wearing sun glasses to avoid any kind of eye contact with the dog, she was wearing blue T-shirt and brown trousers, she did not display any social cues during the test and she did not speak at all. She was moving along the same route as the RC car in the *Mechanical UMO* condition with constant speed (see Figure 2).

### Procedure

#### Familiarization

1. The owner and the dog (on leash) entered the room and walked around. There were three empty bowls, the UMO (at the start point), in the *Mechanic*al and *Social UMO* conditions, or female human in the *Mechanical Human* condition, and the metal box placed at a fixed location; E2 stood in the corner of the lab. The dog could sniff and explore the room on leash for 1 minute. Then the owner sat down at a predetermined location and held the dog in front of him/herself.
2. Experimenter 1 (E1) entered the room and put three pieces of dry food into one of the tree bowls and left the room.
3. The owner took of the leash and encouraged the dog to eat the food (e.g. „It’s yours’; “Come on take it” etc.). After having eaten the food the owner called the dog back. This procedure (Steps 2 and 3) was repeated two times.
4. The UMO or the female human started to move around the room (for 30 sec) in full view of the dog. In the *Mechanical UMO* and *Mechanical Human* conditions they were circling around the bowls travelling on the same path. In contrast, the *Social UMO* moved along varied routes in the room. All partners moved for the same amount of time.
5. Steps 2 and 3 were repeated two times, except that the *Mechanic*al and *Social UMO* or the *Mechanical Human* were moving always in the same way as in Step 4. After the second feeding the partner returned to the start point.

#### Test trials

In Mechanical UMO and Mechanical Human conditions the experiment consisted of 6 trials. One trial consisted of the following steps:

1. E1 entered the room put three pieces of food into one bowl (she baited always the same bowl during the trials), and left.
2. The *Mechanical UMO* or the *Mechanical Human* approached the baited bowl, carried it into the box, left it inside, and returned to the predetermined start point. The bowl was inaccessible for the dogs but they could see it and smell the food.
3. Owner released the dog from the leash, and it was allowed to move freely for 30 seconds. By knocking at the door E1 informs the owner to call back the dog.
4. The *Mechanical UMO* or the *Mechanical Human* returned to the box and brought/took out the bowl, and stopped with it in front of the dog.
5. The owner let the dog eat the food, and the partner returned to the start point.

The *Social UMO* condition consisted of 7 trials. The 1^st^ and the 7^th^ trials were exactly the same as test trials in the *Mechanical UMO* and *Mechanical Human* conditions; including the position of the start point of the partner (see Figure 2).

The 2^nd^ to 6^th^ trials were similar to the 1^st^ and 7^th^ one, except that during Step 1 the experimenter varied the position of the baited bowl, at the end of Step 2 the car stops at various points in the lab (potential start points, see Figure 2) and finally during Step 3 E2 started to move the car toward the box after the dog displayed the first, short (approximately 1 s long) glance at it.

### Behavioural Variables and Data Analysis

All trials were videotaped and dogs’ behaviour during the 30 s of free movement was analyzed later with Solomon Coder 12.06.06 (András Péter http://solomoncoder.com).

*Looking at the partner* (s): looking duration at the partner (UMO or human).

*Latency of looking at the partner* (s): time span from owner releasing the dog until the dog looks first at the partner (UMO or human).

*Latency of touching the partner* (s): time span from owner releasing the dog until the dog touches first the partner (UMO or human) with its muzzle.

*Frequency of gaze alternation*: number looks from the partner (UMO or human) to the box (place of food) directly or vice versa regardless of order.

Inter-observer agreement (between two coders) was assessed by recoding a randomly selected 25% of the subjects (Cohen’s Kappa, 0.98).

For statistical analysis we used IBM SPSS Statistics 21. For the Binary GLMM (for Binomial distribution) we calculated the *Ratio of looking (*number of dogs who looked or did not look) at the partner (UMO or Human) in each trial, and the *Ratio of touching* (number of dogs who touched or did not touch the partner (UMO or Human) with muzzle in each trial.

In the first series of analyses we studied the effect of the repetition, and difference in embodiment and behaviour by comparing the *Mechanical UMO* and *Mechanical Human* conditions. The square-transformed *Looking at the partner* was analyzed by the means of a GLMM (Generalized Linear Mixed Model) for Normal distribution. We analyzed *Ratio of looking/touching dogs* variables with Binary GLMM (for Binomial distribution) to examine whether the subjects looked or did not look at or touched or did not touch the partner (UMO or Human) during the 30 s. Next we analyzed whether there was a difference in the *Latency of touching* the partner between the *Mechanical UMO* and *Mechanical Human* conditions (GLMM for Normal distribution). We also analyzed the *Frequency of gaze alternation* between the partner and the place of food in the two *Mechanical* conditions (GLMM for Poisson distribution).We compared the *Ratio of looking dogs* (with Binary GLMM), and *Latency of looking at the partner* (GLMM for Normal distribution) variables among all the 3 conditions. Finally, we compared all first trials and last trials among all three conditions for all observed behavioural variables (Kruskal-Wallis test with Dunn post-hoc test).

## Results

### Comparison of *Mechanical UMO* and *Mechanical Human* Conditions

**Figure 3:**
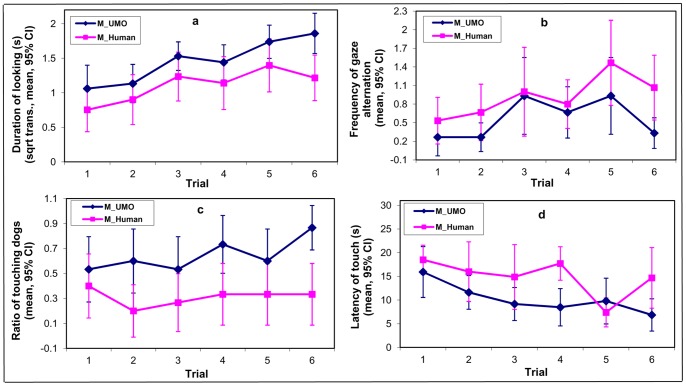
Comparison of different behavioural measures between the *Mechanical UMO* and *Mechanical Human* condition during a 30 sec period in each trial when dogs were allowed to move freely. a; mean duration of looking at the partner (UMO or Human) b; mean frequency of gaze alternations between the partner (UMO or Human) and the place of food c; ratio of dogs who touched the partner with its muzzle (UMO or Human) d; mean latency of touching the partner with muzzle (UMO or Human).

First we compared the two mechanical conditions (*Mechanical UMO* and *Mechanical Human*) to see whether dogs showed comparable behaviour toward the *Mechanical UMO* and the *Mechanical Human*. Dogs in both conditions were looking longer at the partner over repeated trials (F_5,136_ = 7.59, p<0.0001). At the same time dogs looking longer to the *Mechanical UMO* than the *Mechanical Human* (F_1,12_ = 5.37, p = 0.039) (Figure 3/a). Gaze alternations between the partner and the place of food became more frequent with repeated trials in both conditions (F_5,55_ = 3.35, p = 0.01), and on the whole dogs in the *Mechanical Human* condition displayed more gaze alternations than dogs in the *Mechanical UMO* condition (F_1,47_ = 4.5, p = 0.038) (Figure 3/b). More dogs touched the partner in the *Mechanical UMO* condition (F_1,46_ = 10.38, p = 0.002), however this behaviour did not change with the trials (F_5,95_ = 1.02, p = 0.4) (Figure 3/c). Dogs also touched the partner sooner in the *Mechanical UMO* condition than dogs in the *Mechanical Human* condition (F_1,22_ = 4.37, p = 0.048), but this latency did not change with the trials (F_5,17_ = 1.98, p = 0.134) (Figure 3/d).

### Analysis of the Ratio of Looking Dogs and Latency of Looking at the Partner Variables

**Figure 4:**
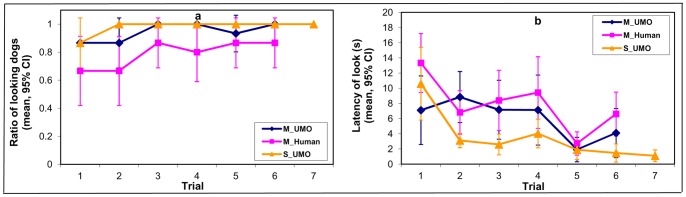
Comparison of the ratio of looking dogs and the latency of looking at the partner in the *Mechanical UMO*, *Mechanical Human* and *Social UMO* conditions during a 30 sec period in each trial when dogs were allowed to move freely. a; ratio of dogs looked at the partner b; mean latency of looking at the partner.

Interactivity of the Social UMO did not allow us to compare most behavioural variables during trials 2^th^ to 6^th^ because the partner started to move when the dog looked at it (see Methods). However, we could analyse how many dogs looked at the partner (*Ratio of looking dogs)* and the latency of this action (*Latency of looking at the partner)*. We found that trials had an effect on how many dogs looked at the partner at all (F_6,39_ = 36.7, p<0.0001) (Figure 4/a). Conditions also differed in the *Ratio of looking dogs* (F_2,8_ = 10.3, p = 0.005). More dogs looked at the partner in the *Social UMO* condition than in the *Mechanical UMO* (p = 0.001) or in the *Mechanical Human* condition (p = 0.033). At the same time fewer dogs looked at the *Mechanical Human* than the *Mechanical UMO* (p = 0.035). In general, dogs looked sooner at the partner as trials went by (F_6,67_ = 10.9, p<0.0001), and condition also had an effect (F_2,46_ = 11.15, p<0.0001). Dogs in the *Social UMO* condition looked first to the partner sooner than dogs in the *Mechanical Human* condition (p = 0.0001), but there were no differences between the two types of UMOs (p = 0.069) or between the two mechanical partners (p = 0.18) (Figure 4/b).

### Comparison of Dogs’ Behaviour in the First and Last Trials

**Figure 5:**
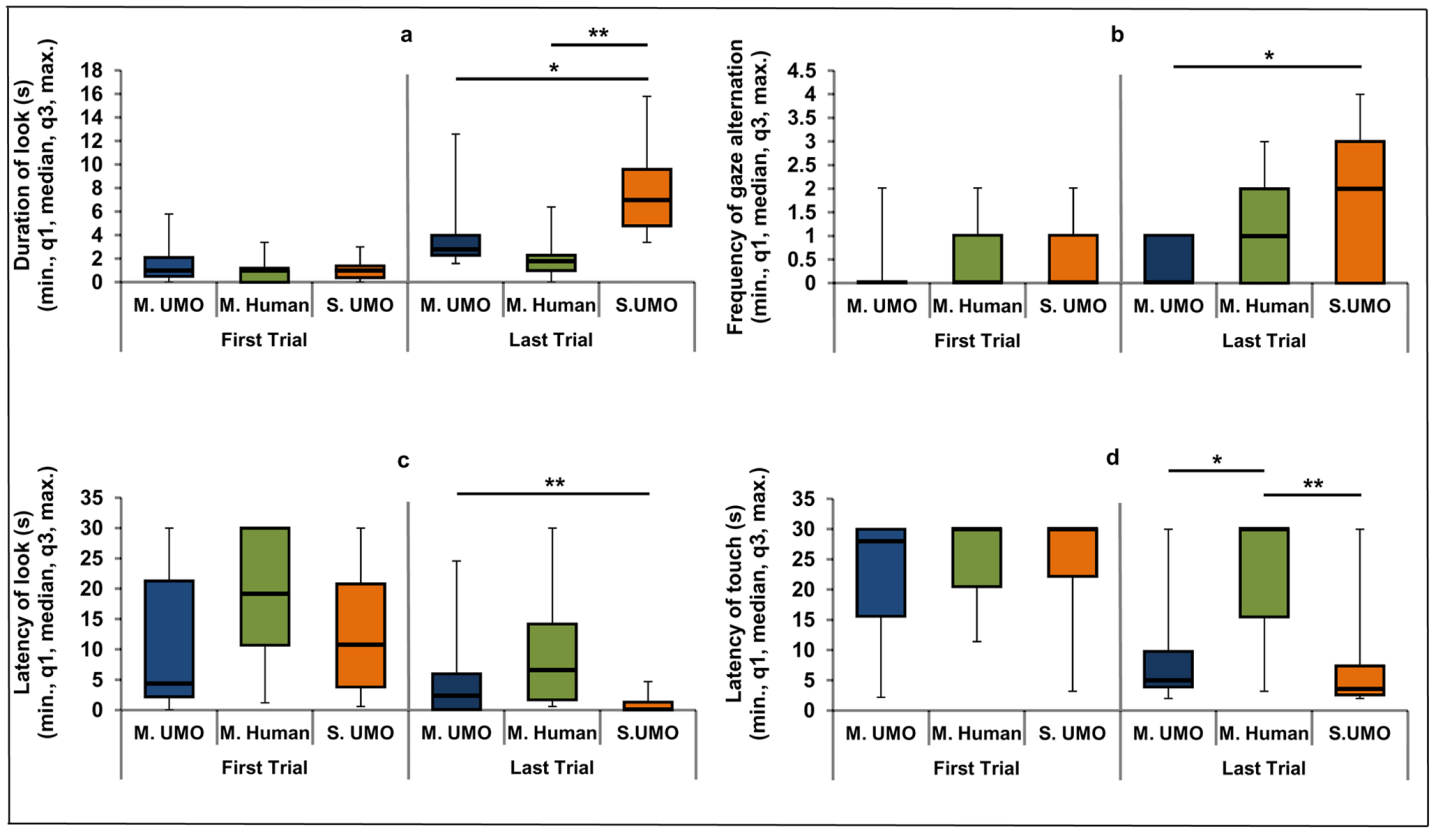
Analysis of the dogs’ behavioural variables during the first and last trials in each condition. a; mean duration of looking at the partner (UMO or Human) b; mean frequency of gaze alternations between the partner (UMO or Human) and the place of food c; mean latency of looking at the partner (UMO or Human) d; mean latency of touching the partner with muzzle (UMO or Human) (* p<0.05, ** p<0.005).

The aim of these comparisons was to examine whether dogs showed more intensive gazing and touching behaviours toward the *Social UMO* than dogs in the mechanical conditions toward the *Mechanical UMO* or the *Mechanical Human*. This effect could emerge as the result of differential type of interactions in trials 2^th^ to 6^th^ (see Methods). In the first trial there were no differences among the three conditions in any of the measured behaviour variables, however during the last trial all variables differed significantly across the conditions (see Table 1). Dogs looked longer at the *Social UMO* than the *Mechanical UMO* or the *Mechanical Human* during the last trial (Figure 5/a). Dogs also altered their gaze more frequently between the *Social UMO* and the place of food during the last trial compared to the *Mechanical UMO*, but no such difference was present in relation the *Mechanical Human* (Figure 5/b). They were also faster to look at the partner in the *Social UMO* condition than in the *Mechanical Human* condition (Figure 5/c). Latency of touching showed the same pattern. Dogs touched the *Social UMO* and the *Mechanical UMO* sooner than the *Mechanical Human* (Figure 5/d).

**Table 1:** Comparison of dogs’ behaviour during the first and last trials of each condition.

Kruskal-Wallis Test, Dunn Post-hoc (N = 47, df = 2)		
Name of the behaviour observed	First trial*	Last trial**
*Looking at the partner*	Chi^2^ = 1.59, p = 0.45	Chi^2^ = 27.46, p<0.0001
		SU vs MU p = 0.008
		SU vs MH p<0.0001
*Frequency of gaze alternation*	Chi^2^ = 1.91, p = 0.38	Chi^2^ = 9.03, p = 0.011
		MU vs SU p = 0.008
*Latency of looking at the partner*	Chi^2^ = 5.61, p = 0.06	Chi^2^ = 15.2, p<0.0001
		SU vs MU p<0.0001
*Latency of touching the partner*	Chi^2^ = 1.04, p = 0.59	Chi^2^ = 11.365, p = 0.003
		SU vs MH p = 0.003
		MU vs MH p = 0.046

* The second column shows the comparison of the first trials among the three conditions; all are non-significant.

** Third column shows the comparison of the last trials, and Dunn’s post hoc comparisons among the conditions (*SU = Social UMO, MU = Mechanical UMO, MH = Mechanical Human*).

## Discussion

The aim of this study was to investigate whether dogs are able to differentiate agents on the basis of their behaviour and show social behaviours toward an UMO (Unidentified Moving Object) if the agent behaves appropriately in an interactive situation. In order to observe such interaction we modelled an experimental situation in which the dog is faced with inaccessible food. Miklósi et al [21] showed that in this case dogs increase their looking time at a human helper and show gaze alternation between the inaccessible food and the human. These observations have been replicated by Gaunet [23] and Horn et al [26], and the authors implicated that the dogs’ behaviour reflects communicative intentions. The present experiment showed that these behaviour features also emerge in the dogs while they are interacting with an UMO, moreover the onset of these behaviours is facilitated by the social features of the UMO: Dogs look longer and show more gaze alternation if the UMO carries eyes, shows variations in its path of movement, displays goal-directed behaviour and contingent reactivity (reacts to the looking action of the dog by retrieving the inaccessible food item). The similarity in the dogs’ behaviour toward the human (in [21]) and the UMOs in the present experiment leads to a range of interesting statements.

First, in order to control for the embodiment we included also a “mechanical human” who looked very differently from the UMO but showed very similar gross movements to the *Mechanical UMO*, e.g. moved along the same path and did not show contigent reactivity to the dog. Naturally, the human used the hand to handle the food. Despite the fact that dogs probably recognised the human in terms of embodiment they were attracted much less to the human as dogs looked longer and touched sooner the *Mechanical UMO* than the *Mechanical Human* (see Figure 3). This could be explained by the fact that dogs have never met the UMO before, and therefore they did not have any expectations about the behaviour of this moving object. Moreover, their previous experience with typical humans may have induced some wariness toward the *Mechanical Human* that manifested in shorter looking and touching duration but in increased gaze alternation.

Second, dogs show a drop in gaze alternation after the penultimate trial (5^th^) toward both mechanical partners but not toward the *Social UMO* (Figure 4b). Although the nature of this phenomenon is unclear, we suggest that dogs have changed their behaviour strategy toward these agents. The increase in looking time and gaze alternation frequency may have been caused by the dogs’ tendency to generalise their previous experience with humans in such situations. Thus they may have recognised the correspondence between their earlier experience and the present situation despite the fact that the UMO is strikingly different from a human. Accordingly, this drop may indicate that dogs gave up showing communicative behaviours toward the agent, and instead “waited” until the agent solved the problem. This is also supported by the observation that such drop did not emerge in the case of the *Social UMO* that replicated the behaviour of a typical human partner under these conditions.

Third, in the present experiment we did not want to account for all possible social features that may facilitate the interaction between the dog and the UMO. Thus the *Social UMO* displayed morphological (eyes spots), motor (travelling along divergent paths) and interactive (starting to move upon being gazed at) characters which made it appear more animate and social at the same time. Despite all these differences the dogs’ behaviour was very similar toward all partners in the very first trial (Figure 5) (although they had the opportunity to observe these agents during the familiarization phase), but changed over repeated interactions. This indicates that the presence of the physical features, like eye spots and varied movements were not the key components for dogs in the case of the *Social UMO*. Instead, goal directedness and interactivity that was displayed in the first and subsequent encounters played a key role in the development and maintenance of social behaviours. These properties of the agent were found to be also significant in allowing human infants to discriminate animate-inanimate displays [27], [28].

Decreased latency of looking at the *Social UMO* can be explained by the fact that it started to move when the dog glanced at it once. Such contingency could emerge quickly in the case of associative learning which has been recently implicated in the development of ‘sense of agency’ (for a review see [29]). Indeed, interaction between social beings (including human infants and caretakers etc.) are accompanied by such forms of learning. The present study looks more at the ‘emergent’ behaviours which could be regarded as ‘by products’ of this contingency and which make the interaction appear more social. Thus we find it interesting that in parallel with dogs’ increased looking behaviour other social behaviours (e.g. touching, gaze alternation) occurred toward the *Social UMO* more often than toward the *Mechanical UMO*.

Interestingly, in another study dogs seemed not to show much social interest toward dog-like robot (AIBO) despite close morphological similarity [17]. Although there are also parallels between the general behaviour pattern of AIBO and the dog, during the interactions the robot did not show any direct reactions to initiative behaviours of the dogs. This also suggests that, not denying the importance for certain morphological features (cf. sign stimuli) in releasing social behaviour, the interactive character of the behaviour on the part of the robot (or in our case the UMO) is more important for evoking social responsiveness than the embodiment per se.

At present most researchers aim to use robots that resemble the studied species as closely as possible (e.g [13]). Although such an approach is important in the study of the effect or morphological and behavioural features in different situations, our findings highlight that the use of UMOs could have several advantages, primarily because this way one can separate the effects of behaviour from the embodiment [6]. This allows the researchers to investigate to what degree the animal is able to deal with the UMO purely on the basis of behaviour displayed.

In human infants the understanding of basic concepts defining the other (e.g. agency, directedness, attention etc.) has been investigated by the means of visual displays showing moving simulated agents in 2D (e.g. [30]). After being habituated to certain events, infants are confronted with unexpected, unnatural events, and researchers deduce the infants’ ability of representing these specific concepts by noting increase in looking time at the time of change (‘surprise effect’, see [31] for a review). Although it is possible to apply the method to some species of animals but there are also methodological problems with measuring eye movement. Thus it would be more advantageous to use real 3D situation to test for similar mental skills in non-human species. We believe that the systematic use of UMOs offers this possibility.

Moreover the use of UMOs could also help answering the question of how much of the social skills are grounded in the species’ embodiment, that is, whether animals are able to represent and deal with social behaviour independently from the body displaying it. Previous social experience makes testing of such sociocognitive abilities difficult among conspecifics, but the unfamiliarity to UMOs and the possibility to use wide range of embodiments make such investigations possible. For example, interaction with UMOs could help in discerning the mental mechanisms related to different forms of social learning [32].

The use of UMOs can also expand the comparison of sociocognitive skills in different species. The comparison of behavioural data collected within a species is often difficult because there are many possible factors that could account for the observed differences [33]. The use of UMOs, which are unfamiliar to all participants that, however, behave in a certain way, could offer a potential way to study the differential capacities of species to interact socially. If the UMOs are deployed in a systematic way (varying their social behaviour) then flexibility of social behaviour across different contexts could also be revealed.

Dogs are especially good candidates for being studied in this way. They are living and have been selected for living in a relationship with humans whose embodiment and behaviour is very different. Despite this divergence dogs and humans are able to develop complex communicative and cooperative interactions [34]. At the moment we do not know to what extent dogs rely on general behavioural homologies present in the social behaviour of both species, and to what degree they extend this basic understanding by learning through everyday experience. Future experiments could reveal the ability of dogs to generalise across contexts and agents, and whether this ability is species specific or emerges as a result of exposure to humans.

## Supporting Information

Video S1 **Procedure.** The video illustrates the procedure of the familiarization and test trial including movements and behaviour of the partner in the *Mechanical UMO*, *Mechanical Human* and *Social UMO* conditions.
(AVI)
